# Study on the Preparation and Chemical Structure Characterization of Melanin from *Boletus griseus*

**DOI:** 10.3390/ijms19123736

**Published:** 2018-11-23

**Authors:** Qiuming Liu, Junjiang Xiao, Bingtong Liu, Yongliang Zhuang, Liping Sun

**Affiliations:** Yunnan Institute of Food Safety, Kunming University of Science and Technology, No. 727 South Jingming Road, Kunming 650500, China; kgqml2012@163.com (Q.L.); 18469121639@163.com (J.X.); liubingtong0126@163.com (B.L.)

**Keywords:** *Boletus griseus*, melanin, chemical structure, mass spectrometry

## Abstract

In this study, melanin (BgM) was obtained from *Boletus griseus*. The chemical composition and structure of BgM were characterized by UV-visible absorption spectrum, Fourier transform infrared spectrum, elemental analysis, nuclear magnetic resonance, pyrolysis gas chromatography mass spectrometry, and ultra-performance liquid chromatography–high resolution mass spectrometry. The percentage contents of C, H, N, S and O elements were 56.38%, 5.86%, 6.17%, 2.44%, and 28.04%, and the S/N and C/N ratios were 0.17 and 10.66, respectively. The UV-vis spectrum of BgM showed a maximum absorption peak at 214 nm. Characteristic absorption peaks were observed at 3426, 1600 and 1105 cm^−1^, and BgM contained phenolic hydroxyl, amidogen, carbonyl, methylene, and methyl groups. Moreover, BgM is an eumelanin, and its main skeleton has both a benzene ring and an indole, and the branched chain mainly consists of alkanes, alcohols, and fatty acids. BgM was hydrolyzed by H_2_O_2_ and four compounds were tentatively analyzed from the UPLC-MS/MS profile. The chemical structure of BgM was characterized as 5,6-dihydroxyindole eumelanin, and the condensed molecular formula is [C_28_(OR_1_)_4_(OR_2_)_3_H_11_O_6_N_4_]_n_.

## 1. Introduction

Melanin is a class of complex polyphenolic heteropolymers and has three main types, namely, eumelanin (brown–black melanin), phaeomelanin (brown-red melanin), and allomelanin (nitrogen-free melanin) [1]. This classification is based on the chemical composition and monomer subunit structure of melanin. Eumelanin and phaeomelanin are derived from dopaquinone which is a common precursor generated by oxidation of tyrosine of tyrosinase. Cysteine or glutathione reacts with dopaquinone and this reaction results in the generation of a variety of cysteinyl-dopa complexes and the formation of benzothiazine intermediates for phaeomelanin formation [2].

Melanin is widely found in bacteria, fungi, animals, and plants. They are considered as bio-organic conductors with distinctive physicochemical properties, and thus have a wide variety of roles. They serve as photoprotectants, pigments, charge transport mediators, free-radical scavengers, and antioxidants [3,4]. Therefore, they are attractive materials that have wide industrial applications in medicine, pharmacology and cosmetics.

However, the basic function of melanin is still a matter of debate and speculation. This uncertainty results from the poorly defined structural and physical–chemical properties of melanin. The chemical composition and structure of melanin from different sources have many uncertainties, with regard to its paramagnetic properties, amorphous nature, chemical insolubility, and complex structure diversity [5,6,7]. Hence, melanin resources remain underdeveloped and underused.

In recent years, several types of melanin from various fungi, such as *Auricularia auricular* [8], *Mycosphaerella fijiensis* [9], *Aspergillus fumigates* [10], *Gliocephalotrichum simplex* [11], and *Lachnum* YM404 [12], have been studied. *Boletus griseus* is one of the most common large fungal species in Yunnan, China. It has a dark color, a long growing period, large output, and low price. The composition and nutritional value of *B. griseus* were evaluated in our previous study [13,14]. However, few studies on the melanin of *B. griseus* have been reported. In the present study, melanin from *B. griseus* was extracted and purified. Then its chemical composition and structure were characterized by UV-visible (UV-vis) absorption spectroscopy, Fourier transform infrared (FTIR) spectroscopy, elemental analysis, nuclear magnetic resonance (NMR), pyrolysis gas chromatography mass spectrometry (Py-GC/MS), and ultra-high performance liquid chromatography-high resolution mass spectrometry (UPLC-MS/MS). This research could provide both theoretical and scientific evidence for further research of natural melanin structure derived from fungi, and also provide a new method for the development and utilization of wild edible fungi in Yunnan.

## 2. Results and Discussion

### 2.1. Verification of BgM

**Figure 1:**
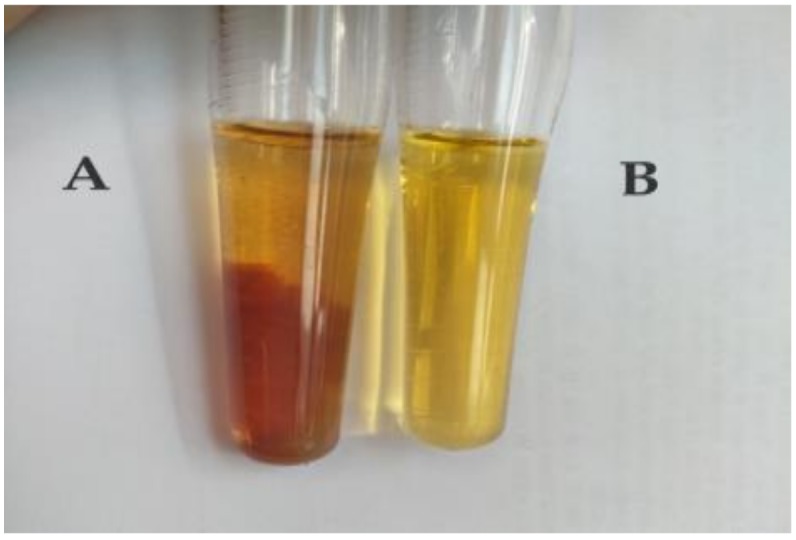
Reaction between BgM and FeCl_3_ solution. (**A**) Reaction between *B. griseus* (BgM) and FeCl_3_ solution (BgM (10 mg) was dissolved in 1 mol/L NaOH solution, and slowly added with FeCl_3_ solution); (**B**) FeCl_3_ solution.

BgM was extracted from *B. griseus* via alkaline extraction and purified via acid hydrolysis and organic solvent treatment. The melanin verification was carried out, as shown in in Figure 1. BgM solution was added to the FeCl_3_ solution, and a large amount of reddish-brown flocculent precipitate appeared immediately. With the continuous addition of the FeCl_3_ solution, the floc gradually disappeared, finally dissolving completely, giving a brownish–yellow solution. This phenomenon was consistent with the previous study, which indicated that melanin had significant reduction-oxidation properties and precipitated in the presence of alkaline FeCl_3_ solution [15,16].

### 2.2. Solubility of BgM

BgM was slightly soluble in 0.1 mol/L NaOH and alkaline methanolic solution (pH 12), soluble in 1 mol/L NaOH, and insoluble in other common organic reagents (acetone, methanol, 75% ethanol, anhydrous ether, petroleum ether, ethyl acetate, and chloroform), and aqueous solutions. It was precipitated in acid solutions including 0.1 mol/L HCl, 1 mol/L HCl and acidic methanolic solution (pH 2.0). The solubility of melanin is related to the pH of the solution. The solubility of melanin was greater with increasing pH value. Our results were consistent with the natural and synthetic melanin solubility in alkaline solution and insolubility in acidic solutions and common organic reagents [17].

### 2.3. Element Analysis of BgM

Element analysis can provide the main elements of melanin, generally C, H, N, S, O, which is an important method for the initial identification of the melanin type [7]. The elemental analyses of BgM are listed in Table 1. The percentages of C, H, N, S, and O were 56.38%, 5.86%, 6.17%, 2.44% and 28.04%, respectively. Compared with the S content of eumelanin (0.09%) and phaeomelanin (9.78%) [18], the S content of BgM was 2.44%, which was higher than that of eumelanin and much lower than that of phaeomelanin. The S/N_(mol/mol)_ of BgM was 0.17. These results indicated that BgM could be classified as eumelanin, with traces of phaeomelanin. Previous research showed that the content of N in 5,6-dihydroxyindole eumelanin is 6% to 9%, which was consistent with our study. In addition, compared with the known eumelanin, BgM contained a lower N content and a higher C/N_(mol/mol)_, indicating that the BgM might have a relatively higher content of fatty substances.

### 2.4. Spectral Properties of BgM

#### 2.4.1. UV-Vis Spectrum

**Figure 2:**
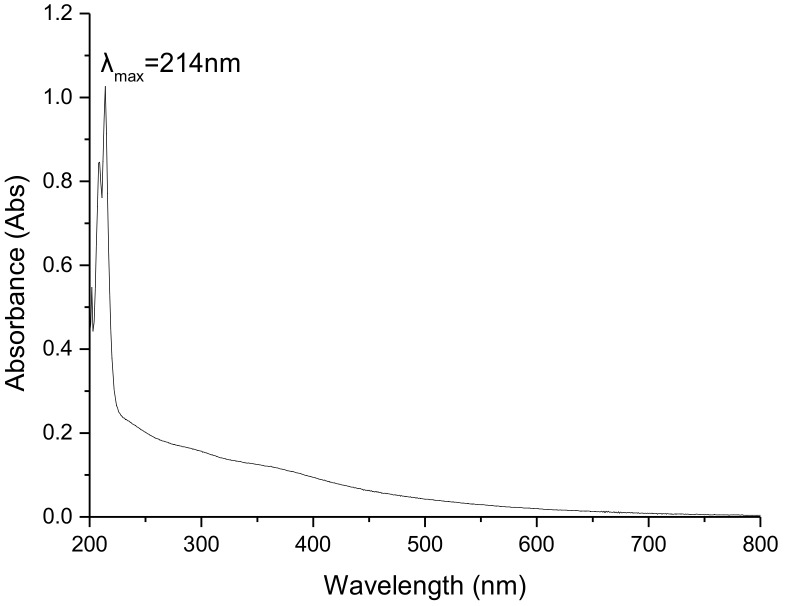
UV-visible (UV-vis) spectrum of (BgM).

As shown in Figure 2, the maximum absorption wavelength of the UV-vis spectrum of BgM was at 214 nm and its optical density gradually decreased with an increase of wavelength. This result was consistent with the previous study [20], which demonstrated that the BgM molecule might have a conjugated double bond system, or an aromatic ring structure. Moreover, an absorption peak was not observed at 260 and 280 nm, showing that BgM did not contain nucleic acid or proteins [21].

#### 2.4.2. FTIR Analysis

**Figure 3:**
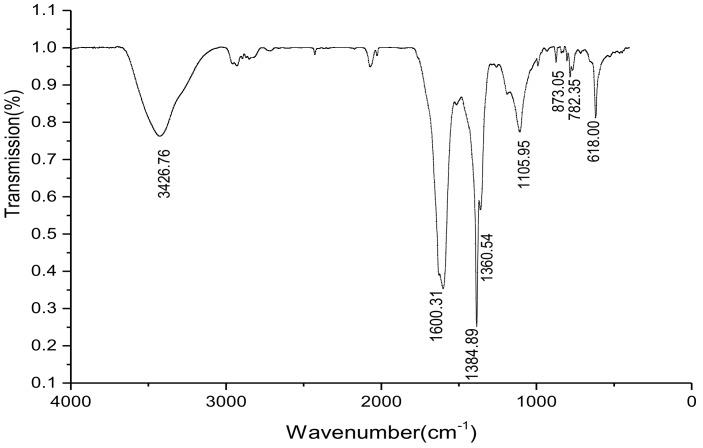
FTIR of BgM.

FTIR is one of the important methods for studying melanin and can obtain information about the major functional groups in the melanin structure [22]. Melanin is a macromolecule with a complex structure and has an infrared spectrum of a series of broad and strong absorption peaks. Each broad peak is formed with many functional groups [23]. As shown in Figure 3, a strong and broad characteristic absorption, corresponding to the stretching vibration of the –OH and –NH_2_ groups [24], was observed at 3426 cm^−1^. In addition, a broad band corresponding C=O stretch [25] was exhibited at 1600 cm^−1^. Obvious characteristic absorption peaks were observed at 3426 and 1600 cm^−1^, which was consistent with the typical melanin characteristic infrared absorption spectrum. An absorption peak was also observed at 1384 cm^−1^, which might be assigned to the stretching vibration of C–CH_3_ [10]. The absorption peak at 1105 cm^−1^ represented an asymmetric stretching vibration of the C–O–C bond. The weak absorption peaks at 618–873 cm^−1^ indicated that the aromatic ring was substituted, forming a conjugated system, and the aryl hydrogen was relatively reduced [26].

#### 2.4.3. ^1^H and ^13^C NMR Analysis

**Figure 4:**
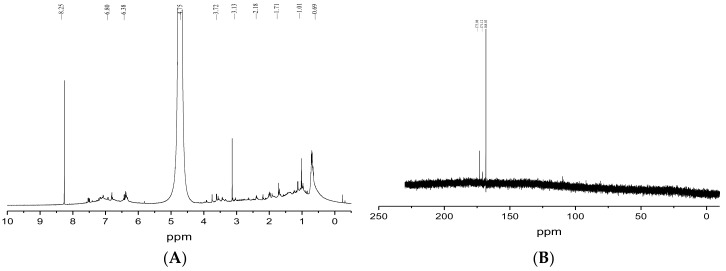
^1^H NMR (**A**) and ^13^C NMR (**B**) of BgM.

The ^1^H NMR spectroscopy of BgM is shown in Figure 4A. In the aliphatic region of the ^1^H NMR spectrum, the signal peak in the range of 0–2.5 ppm could be assigned to the C–H stretching vibration signal peak of the alkyl fragment, which could come from residual proteins [8]. The peaks from 3.2 to 4.2 ppm could be assigned to the –CH_2_– or –CH_3_ group attached to nitrogen and/or oxygen atoms [11,27]. The signal peak in the range of 4.2 to 5.4 ppm was caused by a C=C–H group linked by nitrogen and/or oxygen atoms [28]. The signals in the range of 5.5–6.5 ppm suggested an –NH group of indoles. The wide signal centered on 6.5 to 7.5 ppm was the aromatic hydrogen of the indole or pyrrole ring, which showed the chemical environment around the aromatic hydrogen and the connection diversity among various groups in the BgM. The signal peak in the range of 8.0–8.5 ppm was signed to the –OH group on the ring [27]. The ^13^C NMR spectroscopy date of BgM is shown in Figure 4B, of which obtained signals were few. The main signals appeared in the range of 165–175 ppm, which might be assigned to the back-bone carbonyl groups of peptidic bonds and the side-chain carboxyl and amide groups, as well as the carbonyl group of the quinone moieties of melanin [11].

### 2.5. Py-GC/MS Analysis

Py-GC/MS could provide new information about melanin structure, especially distinguishing between eumelanin and phaeomelanin [29]. The pyrolysis products of BgM are shown in Table 2. Results showed 55 compounds, including toluene, styrene, phenol, phenylacetonitrile, indole, 1-methyl-1H pyrrole, and small amounts of pyridine and hydrocarbons. According to the chemical composition, the major BgM pyrolysis products could be divided into the following groups: Benzene (2, 6, 10, 11, 12, 15, 16 and 18), indole (37, 40, 42, 43 and 44), pyrazole (1 and 3), phenol (17, 24, 25, 28, 30 and 33), furan (7, 13 and 31), phenyl nitrile (20 and 29), pyrrole (9 and 14), pyridine (5 and 8), and quinoline (34 and 36). The most abundant products were benzene and its derivatives, followed by indole and its derivatives. Benzene, phenol, indole, pyrrole and their derivatives are the most prominent characteristic thermal degradation products of eumelanin [30]. Furthermore, few quinoline and isoquinoline molecules were detected; these molecules are the characteristic thermal degradation products of phaeomelanin [31]. A previous study [32] has shown that the degradation of indolequinone could produce pyrrole, benzene, and its alkyl derivatives during pyrolysis, and isoquinoline may produce pyridine and its alkyl derivatives. This result indicated that these compounds arose not only from melanin structure but also from the degradation of other polymeric subunits. Moreover, a small amount of phenyl nitrile, furan, and pyrazole were discovered in BgM, which was similar to those studied for natural melanin [29]. Based on the results above, BgM was further confirmed to consist of a large amount of 5,6-dihydroxyindole eumelanin and a small amount of phaeomelanin, and the basic skeleton is benzene ring and indole. Furthermore, the pyrolysis products also contained hydrocarbons (39, 41, 46, 47, 48, and 49), alcohols (4 and 45), and fatty acids (50, 51, and 52), which should mainly be the branched chains of BgM. This result was consistent with the higher C/N_(mol/mol)_, which was measured in the element analysis (Table 2).

### 2.6. UPLC-MS/MS Analysis

**Figure 5:**
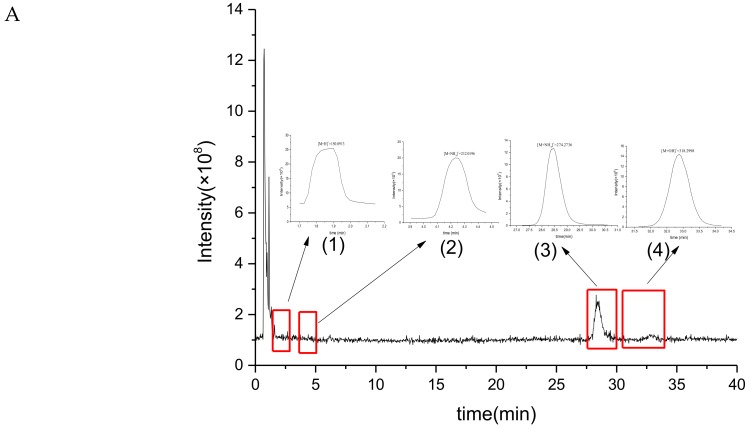

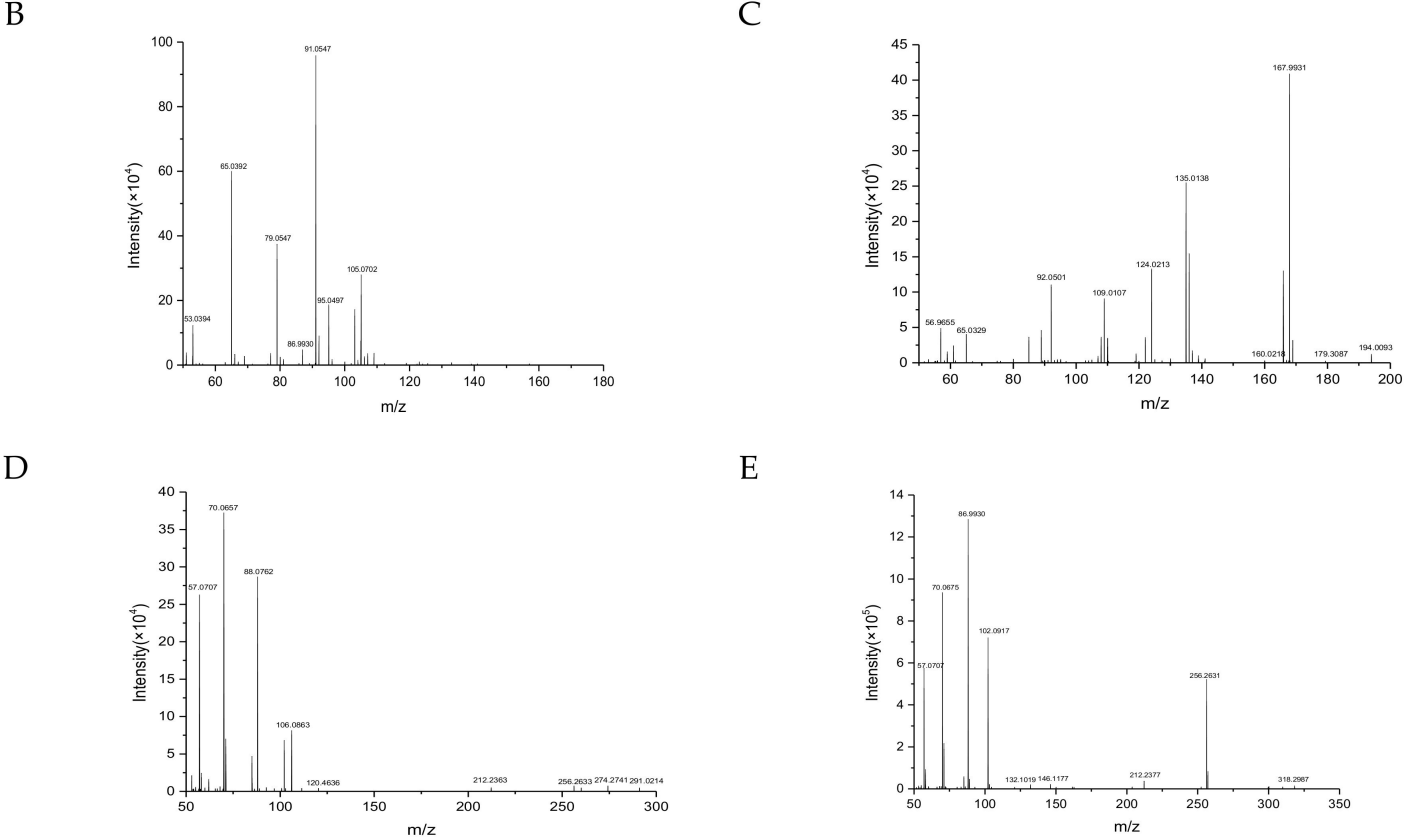
UPLC-MS/MS of oxidative degradation product BgM1. (**A**) The total ion chromatogram of UPLC-MS/MS of BgM1 ((1), (2), (3) and (4) was the primary mass spectrum of peak 1, 2, 3 and 4, respectively), and the red frames were the range of peaks 1, 2, 3 and 4, respectively; (**B**) MS^2^ ion fragment of mass spectrum peak 1; (**C**) MS^2^ ion fragment of mass spectrum peak 2; (**D**) MS^2^ ion fragment of mass spectrum peak 3; (**E**) MS^2^ ion fragment of mass spectrum peak 4.

UPLC-MS/MS analysis can speculate the molecular mass and possible molecular structure of the unknown compound via the information obtained by the ion fragments. Therefore, UPLC-MS/MS analysis could be carried out to further confirm the structure of BgM. Chemical degradation methods are often used to analyze the structure of melanin, due to the insolubility of melanin in water and most organic solvents. In our case, BgM was degraded via H_2_O_2_ oxidation to obtain the soluble degradation product BgM1. The UPLC-MS/MS analysis was performed to further obtain the structural information of BgM. The total ion chromatogram of UPLC-MS/MS of BgM1 was analyzed using MZmine 2.32 and Thermo Xcalibur Qual Browser 3.0 software, and four main mass spectral peaks were identified (Figure 5).

The quasi-molecular ion peak of mass spectrum peak 1 was [M + H]^+^ *m*/*z* = 150.0913, and its relative molecular mass should be 149 (Figure 5A). The MS^2^ main ion fragments 53.0394, 65.0392, 79.0547, 86.9930, 91.0547, 95.0497, and 105.0702 were obtained, for which the molecular formula was speculated to be C_8_H_7_O_2_N (Figure 5B).

The quasi-molecular ion peak of mass spectrum peak 2 was [M + NH_4_]^+^ *m*/*z* = 212.0196, and its relative molecular mass should be 193 (Figure 5A). The MS^2^ main ion fragments 56.9655, 65.0392, 92.0501, 109.0107, 124.0213, 135.0138, 160.0218, 167.9931, 179.3087, and 194.0093 were obtained, for which the molecular formula was speculated to be C_9_H_7_O_4_N (Figure 5C).

The quasi-molecular ion peak of mass spectrum peak 3 was [M + NH_4_]^+^ *m*/*z* = 274.2736, and its relative molecular mass should be 256 (Figure 5A). The MS^2^ main ion fragments 57.0707, 70.0657, 88.0762, 106.0863, 120.4636, 212.2363, 256.2633, 274.2741, and 291.0214 were obtained, for which the molecular formula was speculated to be C_13_H_8_O_4_N_2_ (Figure 5D).

The quasi-molecular ion peak of mass spectrum peak 4 was [M + OH]^+^ *m*/*z* = 318.2998, and its relative molecular mass should be 300 (Figure 5A). The MS^2^ main ion fragments 57.0707, 70.0657, 88.0762, 102.0917, 132.1019, 146.1177, 212.2377, 256.2631, and 318.2987 were obtained, for which the molecular formula was speculated to be C_14_H_8_O_6_N_2_ (Figure 5E).

### 2.7. Structural Analysis of Melanin M

**Figure 6:**
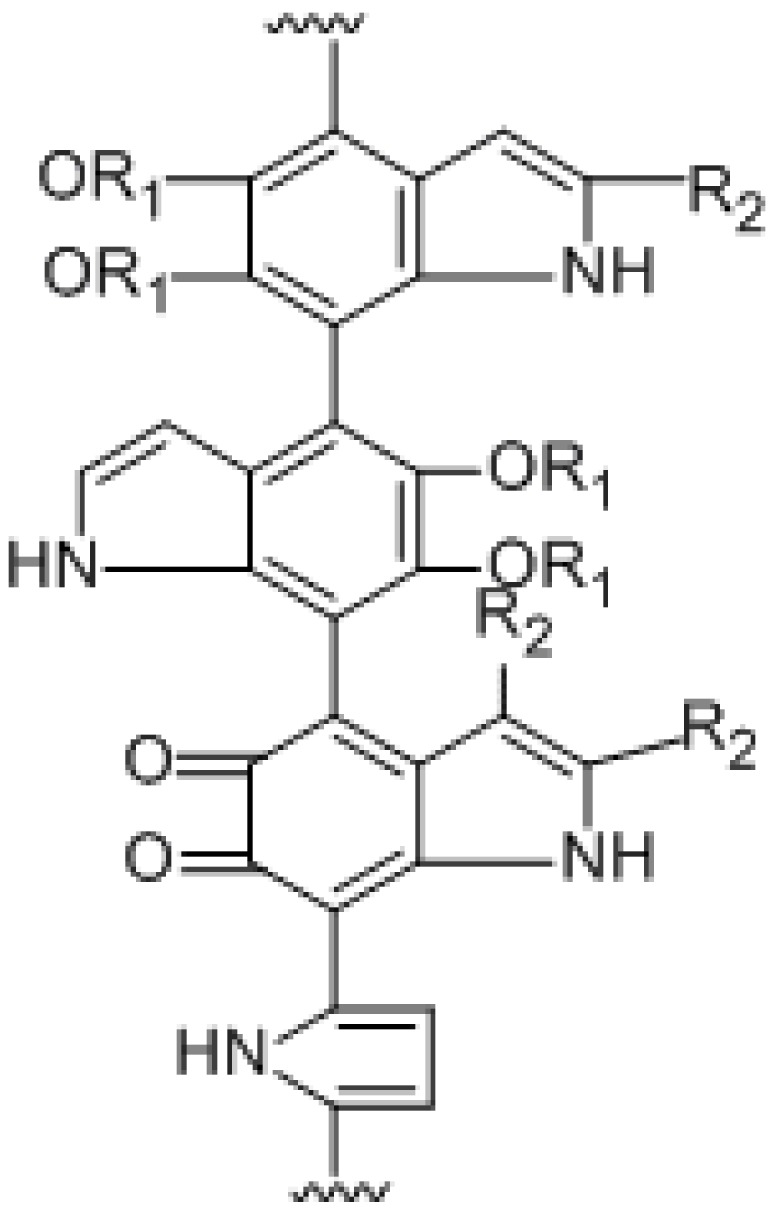
The structure of BgM. R_1_=H/alkanes/alcohols/fatty acid; R_2_=CH_3_/COOH.

Combining the analysis results of FTIR, ^1^H and ^13^C NMR, Py-GC/MS of BgM and the UPLC-MS/MS of oxidative degradation product BgM1, we speculated that the condensed molecular formula of BgM was [C_28_(OR_1_)_4_(OR_2_)_3_H_11_O_6_N_4_]_n_, and the possible chemical structure could be inferred as shown in Figure 6. The structure consisted of 5,6-dihydroxyindole and its derivatives and associated with some alkanes, alcohols and fatty acids, which could be interconnected by various chemical bonds to form a macromolecular polymer.

## 3. Experimental Section

### 3.1. Chemicals

Analytical grade chemicals, including sodium hydroxide, hydrochloric acid, potassium hydroxide, chloroform, isoamyl alcohol, ethanol, acetone, methanol, petroleum ether, ethyl acetate, citric acid ammonia, and hydrogen peroxide were purchased from Nanjing Jiancheng Institute of Biotechnology (Nanjing, China). D_2_O and NaOD were purchased from cambridge isotope laboratories, Inc. Formic acid and acetonitrile were purchased from Merck (Darmstadt, Germany). HPLC grade solvent ammonium formate was purchased from Shanghai Macklin Biochemical Co., Ltd. (Shanghai, China). Ultra-pure water was prepared with laboratory ultrapure water machine (Chengdu You Yue Technology Co., Ltd., Chengdu, China).

### 3.2. Material

The fruiting body of *B. griseus* was purchased from the local market of Yunnan province, China. The surface of the fresh fruiting body was removed and washed successively with running water and ultrapure water. The cleaned samples were sliced, freeze-dried, crushed, and passed through a 40-mesh sieve. The sample powder was stored in sealed polyethylene bags under dry and dark conditions for further experiments.

### 3.3. Melanin Preparation

Melanin was extracted from *B. griseus* according to previous methods [32] with some modifications. Briefly, 3 g of the sample powder was added 90 mL 1 mol/L NaOH, and then incubated at 60 °C for 40 min. The mixture was centrifuged (10,000 rpm, 10 min) and the supernatant was collected. The pH value was adjusted to 1.5 with 6 mol/L HCl, and the supernatant was heated in a water bath at 80 °C for 12 h, followed centrifugation at 10,000 rpm for 10 min. The precipitate was collected and washed with deionized water until the supernatant was colorless. The crude melanin was obtained through vacuum freeze-dry. Furthermore, the crude melanin was purified. Crude melanin (2 g) was hydrolyzed with 10 mL 7 mol/L HCl solution and boiled for 4 h. The precipitate was collected via filtration and washed with ultrapure water thrice. The precipitate was dissolved in 10 mL 1 mmol/L KOH and added with 10 mL chloroform: Isoamyl alcohol (1:5). The mixture was centrifuged (10,000 rpm, 10 min). This process was repeated thrice. The pH of the precipitate was adjusted to 2.0 using 1 mol/L HCl. The suspension was centrifuged (10,000 rpm, 10 min) and the precipitate was collected. Then, the precipitate was washed successively with different concentrations of ethanol (100%, 95%, 75%) and ultrapure water. Finally, the precipitate was collected, vacuum freeze-dried to obtain melanin of *B. griseus* (BgM), and stored at −20 °C.

### 3.4. Melanin Verification

BgM (10 mg) was dissolved in 1 mol/L NaOH solution and slowly added with a FeCl_3_ solution. The phenomenon was observed and noted.

### 3.5. Melanin Solubility

BgM (5 mg) was added with acetone, methanol, 75% ethanol, absolute ethanol, petroleum ether, ethyl acetate, chloroform, ultrapure water, 0.1 mol/L citric acid solution, 0.1 mol/L HCl, 1 mol/L HCl, 0.1 mol/L NaOH,1 mol/L NaOH, acidic (pH 2.0), and alkaline (pH 12.0) methanolic solution. The dissolution was observed after standing for 30 min.

### 3.6. Elemental Analysis

The percentage contents of C, H, N, S, and O in BgM were measured using a vario MICRO cube element analyzer (Elementar Analysensystem GmbH, Hannover, Germany).

### 3.7. Assay of Spectral Properties of BgM

#### 3.7.1. UV-Visible Absorption Spectrum

BgM was dissolved in 1 mol/L NaOH at a final concentration of 0.25 mg/mL. The UV-vis spectrum was recorded from 200 to 800 nm using a UV-Vis spectrophotometer (PERSEE, Beijing, China), and 1 mol/L NaOH solution was used as the reference.

#### 3.7.2. Fourier-Transform Infrared Spectrum

The mixture of BgM (1.5 mg) and dry KBr (200 mg) were pressed into tablets, and the FTIR spectra were measured between 4000–400 cm^−1^ using a Magna-IR 750 FTIR spectrometer (Thermo Nicolet Co., Waltham, MA, USA).

#### 3.7.3. Nuclear Magnetic Resonance

BgM was dissolved using the solvent D_2_O/NaOD, and the ^1^H spectrum and the ^13^C spectrum of BgM were recorded on a Bruker-400NMR spectrometer (Bruker Co., Leipzig, Germany).

### 3.8. Pyrolysis Gas Chromatography Mass Spectrometry

BgM was cleaved via single-step lysis by Frontier EGA/PY3030D thermal cracking apparatus (Frontier, Fukushima, Japan), analyzed by GCMS-QP2020 (SHIMADZU, Kyoto, Japan), and qualitatively searched using the National Institute of Standards and Technology (NIST) standard library.

The analysis conditions were as follows: The pyrolyzer was set at 650 °C and the pyrolysis time was 18 s. The GC/MS analytical column was Rxi-5Sil MS 30 m × 0.25 mm × 0.25 µm. The GC oven temperature was operated from 50 °C (isothermal for 2 min) to 260 °C at a rate of 5 °C/min, and then isothermal for 8 min. The GC injector was maintained at 250 °C. The constant line speed was 36.3 cm/s and the split ratio was 40/1. The ionization method was EI. The ionization source temperature was 230 °C. The acquisition method was “scan”, and the mass range was *m*/*z* 45–500 amu.

### 3.9. Ultra High Performance Liquid Chromatography-High Resolution Mass Spectrometry

BgM was degraded by H_2_O_2_ oxidation. BgM was dissolved in 5.8 mL 5% H_2_O_2_ and the pH was adjusted to 10.7 with ammonia. The mixture was under ultrasonic conditions, as follows: 70 °C, 40 KHZ for 10 min, and then freeze-dried to obtain degradation product BgM1.

The mass spectrum of BgM1 was analyzed by UPLC-MS/MS (Thermo Fisher Scientific, USA). The chromatographic conditions were as follows: Column: Poroshell 120 EC-C_18_ 2.1 × 100 mm 1.9 µm; the mobile phase used was a 70:30 (*v*/*v*) mixture of 5 mmol/L ammonium formate, containing 0.1% formic acid, and acetonitrile; column temperature: 40 °C; flow rate: 0.3 mL/min; injection volume: 3 µL; and the elution isocratic was 60 min.

The mass spectrometry conditions were as follows: Positive ion detection mode (ESI^+^), scan range *m*/*z* 100–1000, dryer temperature 350 °C, dryer flow rate 10 L/min, nebulizer pressure 40 psi, capillary outlet voltage 4000 V, and multi-reaction monitoring Mode (MRM).

### 3.10. Statistical Analysis

The experimental results of the element analysis were expressed as means ± SD of triplicates. The qualitative research of Py-GC/MS was performed using the National Institute of Standards and Technology (NIST) standard library. UPLC-MS/MS was analyzed using MZmine 2.32 and Thermo Xcalibur Qual Browser 3.0 software.

## 4. Conclusion

The BgM was prepared from *B. griseus*, and its chemical composition and structure was determined through several analyses. The results showed that the melanin from *B. griseus* was mainly composed of 5,6-dihydroxyindole and its derivatives. Its condensed molecular formula ([C_28_(OR_1_)_4_(OR_2_)_3_H_11_O_6_N_4_]_n_) and structure formula were also concluded. To our knowledge, this study is the first to identify the molecular and structural formulas of melanin derived from *B. griseus*. This study could promote the development and utilization of fungal melanin.

## Figures and Tables

**Table 1:** Element analysis of BgM.

Element	BgM (%)	Eumelanin * (%)	Phaeomelanin * (%)
C	56.38 ± 0.3	56.45	46.24
H	5.86 ± 0.01	3.15	4.46
N	6.17 ± 0.19	8.49	9.36
S	2.44 ± 0.12	0.09	9.78
O	28.04 ± 0.55	31.82	30.16
S/N_(mol/mol)_	0.17	0.01	0.46
C/N_(mol/mol)_	10.66	7.76	5.75

* These dates were derived from Ito’s [19] study.

**Table 2:** The Py-GC/MS products of BgM.

Number	Compound Structure	Retention Index	Number	Compound Structure	Retention Index	Number	Compound Structure	Retention Index
1	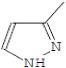	921	20	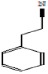	1238	38	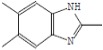	1588
2	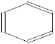	680	21	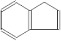	1029	39	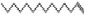	1403
3	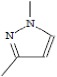	804	22	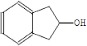	1235	40	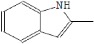	1264
4	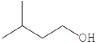	697	23	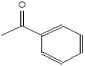	1029	41	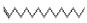	1502
5	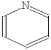	674	24	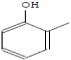	1014	42	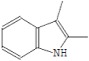	1353
6	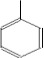	794	25	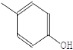	1014	43	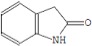	1210
7	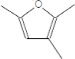	821	26	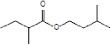	1054	44	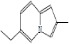	1269
8	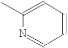	787	27	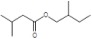	1054	45	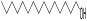	1755
9	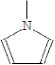	677	28	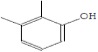	1127	46	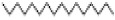	1612
10	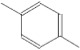	907	29	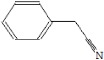	1138	47	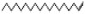	1801
11	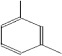	907	30	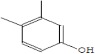	1127	48	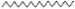	1810
12	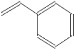	883	31	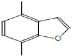	1244	49	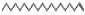	1900
13	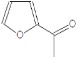	878	32	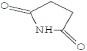	934	50	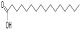	1968
14	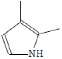	889	33	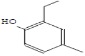	1227	51	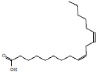	2183
15	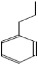	992	34	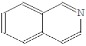	1225	52	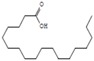	2167
16	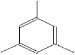	1020	35	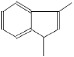	1179	53	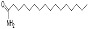	2021
17	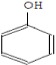	901	36	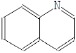	1225	54	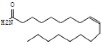	2228
18	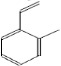	996	37	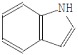	1174	55	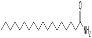	2220
19	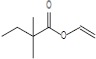	889						
